# *CMAH* Coding Sequence Variants in 15 Non-Domestic Felid Species Related to *ABC* Blood Group System

**DOI:** 10.3390/ani14162442

**Published:** 2024-08-22

**Authors:** Alexandra Kehl, Henrike Kuder, Lily Parkinson, Amie Koenig, Ines Langbein-Detsch, Elisabeth Mueller, Urs Giger

**Affiliations:** 1 Laboklin GmbH & Co. KG, 97688 Bad Kissingen, Germanymueller@laboklin.com (E.M.); 2 Comparative Experimental Pathology, School of Medicine, Technical University of Munich (TUM), 81675 Munich, Germany; 3 College of Veterinary Medicine, University of Georgia, Athens, GA 30602, USA; lily.parkinson@brookfieldzoo.org (L.P.); akoenig@uga.edu (A.K.); 4 Vetsuisse Faculty, Klinik für Kleintiermedizin, University of Zürich, 8057 Zürich, Switzerland; giger@upenn.edu

**Keywords:** cat, polymorphism, transfusion, genotype, phenotype, blood compatibility, blood groups

## Abstract

**Simple Summary:**

Humans and other mammals have different blood group systems. In domestic cats, the *ABC* blood group system is the most important and has been studied a lot, from the physical traits to the genetic level. For wild cats (non-domestic felids), tests have been conducted to identify blood types using different methods, but the genetic markers used in domestic cats could not identify blood types *B* and *C* (*AB*) in wild cats. In this study, the *CMAH* gene of 138 blood samples from 15 wild cat species was sequenced to see if certain genetic variants matched with the *ABC* blood types. A total of 58 different genetic variants were found, including 15 causing changes in the protein sequence. One variant (c.635G>C) was linked with blood type *B* (and *C*) in cheetahs and blood type *B* in cougars. Since cheetahs and cougars are part of the same genera puma, it is unclear whether this variant is a marker for type *B* (or *C*) specifically or is just common in pumas.

**Abstract:**

Different blood group systems have been characterized in people and other mammals. In domestic cats, the *ABC* blood group system plays the most important clinical role and has been investigated extensively—from the phenotype to the molecular genetics. In non-domestic felids, phenotypic *ABC* blood typing has been performed by different methods to detect the antigens, but the four informative *CMAH* markers in domestic cats were not able to identify types *B* and *C* (*AB*) in non-domestic cats. In this study, 138 blood samples from 15 non-domestic (wild) felid species were investigated by *CMAH* exonic sequencing and genotyping for putative variants causing type *B* or *C* (*AB*) and correlation to the respective *ABC* blood phenotype. A total of 58 *CMAH* variants were found, including 15 missense and 43 synonymous *CMAH* variants. One variant (c.635G>C) was concordant with blood type *B* (and *C*) in cheetahs and type *B* in cougars, compared to blood type *A* in all other felid species (lion, tiger, Canada lynx, snow leopard, clouded leopard, serval, jaguar, fishing cat, Pallas cat, bobcat, black footed cat, leopard, and sand cat). Since cheetahs and cougars belong to the genera puma, it could not be determined if the common *CMAH* variant is either a marker for type *B* (or *C*) or is just common in pumas.

## 1. Introduction

Blood group systems and their red blood cell (RBC) antigens have been well characterized from serology to protein and molecular genetic determinants in humans [1]. While many blood group systems have been immunologically identified in domestic and other animals, only a few have been further characterized.

The RBC membrane antigens and molecular genetic variants of the *ABC* (*AB*) blood group system in domestic cats (*Felis catus*) represent the most clinically relevant system, and it has been well characterized [2,3,4]. It is composed of type *A*, *B*, and *C* (also called *AB*) [2]. The enzyme cytidine monophosphate-N-acetylneuraminic acid hydroxylase (CMAH) converts sialic acid N-acetylneuraminic acid (NeuAc, type *B* antigen) to N-glycolylneuraminic acid (NeuGc, type *A* antigen), and type *C* expresses both [5]. Different variants in the *CMAH* gene lead to the apparent loss of or reduction in the regular *CMAH* activity needed for type *A* antigen expression, and thereby, to blood type *B* or *C* [6,7,8]. However, no direct CMAH enzyme activity measurements have been reported in cats with different blood types. The blood type frequencies in domestic cats vary greatly depending on the breed and geographic regions. Blood incompatibilities due to naturally occurring allo-antibodies can result in acute hemolytic transfusion reactions in any *A-B* mismatched red blood cell transfusion and in hemolysis of the newborn, also known as neonatal isoerythrolysis, in type *A* and *C* (*AB*) kittens born to type *B* queens due to the universal presence of strong naturally occurring *anti-A* antibodies [2,9].

Erythrocyte antigens have been immunologically assessed in various non-domestic cat species and type *A*, *B*, and *C* have been detected, whereby the blood type *A* was most common and types *B* and *C* were only observed in a few species [10,11,12]. Various laboratory and point-of-care typing methods were compared in the recent study and, within a non-domestic felid species, the blood type was the same for those individuals tested [10]. While there was strong agreement for those species with type *A*, there was some ambiguity among those between types *B* and *C*. Noteworthy is that all cheetahs and cougars had blood type *B* or *C*, while all other non-domestic felids had type *A*, except for one type *C* Canada Lynx and one type C leopard. Genotypes of the four common *CMAH* variants relevant in domestic cats for blood types *B* and *C*, namely *c.179G>T*, *c. 268T>A*, *c.364C>T*, and *c.1322delT* [7,8,13], were recently examined, but none of the non-domestic felid species showed any of these variants to explain type *B* or *C* (*AB*) [10].

The goal of this study was to sequence the *CMAH* exons and adjacent intronic regions and to identify variants associated with blood types *B* and *C* in different non-domestic felid species from zoos and other institutions in North America.

## 2. Materials and Methods

A total of 138 EDTA blood samples from 15 different non-domestic felid species obtained from 72 institutions in the United States between August 2020 and May 2021 were studied. This included cheetahs (*Acinonyx jubatus*, n = 40), lions (*Panthera leo*, n = 26), tigers (*Panthera tigris*, n = 19), Canada lynx (*Lynx canadensis*, n = 11), snow leopards (*Uncia uncia*, n = 10), cougars (*Puma concolor*, n = 6), servals (*Leptailurus serval*, n = 5), clouded leopards (*Neofelis nebulosa*, n = 4), jaguars (*Panthera onca*, n = 4), fishing cats (*Prionailurus viverrinus*, n = 4), Pallas cats (*Felis manul*, n = 3), bobcats (*Lynx rufus*, n = 3), leopards (*Panthera pardus*, n = 2), and one black-footed cat (*Felis nigripes*) and sand cat (*Felis margarita*).

All non-domestic felids from North America and their EDTA-anticoagulated blood samples used in the current study were part of a recent publication on phenotypic and genotypic *CMAH* analyses [10]. Briefly, blood samples were collected for routine wellness or specific disease examinations from captive non-domestic felids at zoos and other institutions and leftover EDTA blood samples were frozen and shipped from Georgia to Germany for DNA extraction, *CMAH* sequencing, and/or genotyping. Institutional Animal Care and Use Committee approval was obtained for these studies from the University of Georgia (UGA IACUC A 2020 01-006-Y1-A0) and CITES (permit #23US46232E/9). 

DNA was extracted using QIAamp DNA Blood Mini kit and DNeasy Blood and Tissue kit (Qiagen, Valencia, CA, USA). Sanger sequencing of all 16 exons with adjacent intronic regions of the *CMAH* gene was performed on one individual from each species (except Cheetahs, where one individual with blood type *B* and one with suspected blood type *C* (*AB*) were included), as described previously for domestic cats [7]. The *CMAH* sequences of the domestic cat (ensembl ENSFCAT00000078694.1; NCBI EF127684.1) and non-domestic felid species were compared with BLAST (https://blast.ncbi.nlm.nih.gov/Blast.cgi, accessed on 12 April 2024). *CMAH* variants 635G>C correlating with blood type *B* (or *C*) were genotyped in additional wild cats. The impact of identified *CMAH* variants on the enzyme was examined by the prediction tool Ensembl Variant Effect Predictor (VEP) [14], but no actual CMAH enzyme activity measurements were performed.

## 3. Results

A total of 138 EDTA blood samples from 15 different non-domestic felid species were included in the study. Their phenotypic blood types, based upon various immunological methods, were recently published [10], and results indicated that within a non-domestic felid species, the blood type was the same, except possibly in cheetahs. Various laboratory and point-of-care typing methods were compared in that study and phenotypic differences were observed. While there was strong agreement for those species with type *A*, there was some ambiguity among those between type *B* or *C*. Noteworthy is that all cheetahs and cougars analyzed so far had blood type *B* or *C*, while all other non-domestic felids (lion, tiger, Canada Lynx, snow leopard, serval, clouded leopard, jaguar, fishing cat, Pallas cat, bobcat, leopard, black-footed cat, sand cat) had type *A*, except for one type *C* Canada Lynx and one leopard [10].

Exonic and adjacent intronic *CMAH* sequences were obtained from one animal from 15 non-domestic felid species (Table 1). One cheetah and one cougar had blood type *B*, one cheetah had apparent blood type *C*, and all others had blood type *A*. When comparing *CMAH* sequences between non-domestic and domestic felids, a total of 58 exonic *CMAH* variants were observed, including 15 missense and 43 synonymous *CMAH* variants in non-domestic felids, but interestingly no nonsense variants. 

Among these missense variants, nine were found to be present homozygously in only one non-domestic felid species. These felids were all phenotyped as having blood type *A*: 26TT in black-footed cat, 53GG in snow leopard, 155TT, 1237GG, and 1714AA in serval, 161CC and 1157GG in African lion, and 327CC and 1013GG in sand cat. Four of these *CMAH* variants were present in several felid species but did not show any correlation with the observed phenotypic blood type results (50T>C, 131G>A, 160G>A, 1353G>T).

One homozygous *CMAH* variant (635G>C) correlated completely with blood type *B* in cougars and cheetahs (and potential type *C* in cheetahs), while variant 1567A>G was only present in cougars.

The only *CMAH* missense variant (327C>A) which was also previously documented in domestic cats but without having an apparent effect on blood type [7] was found in one sand cat, with type *A*, studied here.

The phenotype–genotype correlation seen for *CMAH* 635G>C in the cheetah and cougar was further investigated by genotyping additional non-domestic felids with available DNA samples from 138 felids (Table 2). The six cougars and three cheetahs with type *B* and all thirty-six cheetahs with presumptive blood type *C* immunologically tested showed the genotype 635CC, compared to ninety-one non-domestic cats of thirteen other felid species with exclusively type *A* blood showing genotype 635GG. The one Canada Lynx and one leopard phenotyped as blood type *C* were also homozygous for 635GG, suggesting a phenotypic typing error or additional non-exonic *CMAH* variants to explain the presumptive type *C*.

The 635G>C variant causes alteration of the amino acid sequence by replacing a glycine to an alanine (p.Gly212Ala). Interestingly, all publicly available *CMAH* sequences of cheetahs and cougars uniformly showed the existence of the same alanine instead of glycine at position 212, whereby all other available amino acid sequences of the other non-domestic felid species had glycine at this position. In some other mammalian species, glycine is replaced by arginine (*Crocuta crocuta*, *Hyaena hyaena*), glutamic acid (for instance, *Sus scrofa*, *Vulpes vulpes*, *Canis lupus dingo*), or lysine (*Elephas maximus indicus*), but their phenotypes regarding the *ABC* blood group system are not available. The amino acid alanine at position 212 lies within a conserved domain named UlaG and is part of the Ulag superfamily (NCBI ABO40434). 

The impact of the 635CC missense variant was analyzed by the Ensembl VEP tool [14] that includes a SIFT prediction, which indicated eight variants with moderate impact. While the 635G>C variant was called tolerant by SIFT, it could still have a deleterious impact. Only the *CMAH* variant 1157A>G was assessed by SIFT as deleterious, but it seemed to be without consequences on the *ABC* blood type, since it was only present in lions and they all had blood type *A*.

## 4. Discussion

The sialic acids N-glycolylneuraminic acid (NeuGc; type *A*) and N-acetylneuraminic acid (NeuAc; type *B*) are two major erythrocytic membrane antigens expressed depending on the functional enzyme cytidine monophosphate-N-acetylneuraminic acid hydroxylase (CMAH) in different animal species and breeds, and in some, this is associated with disease [2,15,16,17,18].

While blood incompatibility reactions due to *A-B* mismatches, such as acute hemolytic transfusion reactions and hemolysis of the newborn, are clinically important problems in domestic cats [5,9], they have not been reported in non-domestic felids in the wilderness and in zoos. Our recent and other phenotypic *ABC* blood typing studies of non-domestic felids found that nearly all non-domestic felid species had type *A*, except cheetahs and cougars, which had type *B* (or *C*), as well as a single Canada Lynx and one leopard, which had type *C* in a recent survey [10]. It is possible that a survey of larger numbers of individuals within each species may yield more cats with type *B* or *C*. However, it is also recognized that the lectin *Triticum vulgaris*, used to detect the *B* antigen, can also agglutinate type *A* erythrocytes at higher concentrations in domestic cats, and thus may have, by some typing methods, falsely caused type *C* instead of type *B* results in non-domestic felids [5,10]. Also, the strength and specificity of *anti-A* and *anti-B* poly- and monoclonal antibodies to detect these erythrocytic antigens varied, which might have affected agglutination reactions and, thus, blood typing results [10]. Therefore, more precise genotyping tools with specific *CMAH* variants would be desirable to accurately assign blood types in domestic cats and non-domestic felids and thereby avoid incompatibility reactions.

The CMAH enzyme is encoded by the *CMAH* gene with 16 exons and 1734 bp open reading frame predicted to encode a protein of 578 amino acid residues in domestic cats. Our exonic and adjacent intronic sequencing of the *CMAH* gene of 15 non-domestic felid species in this study showed the same size and close homology of the *CMAH* gene and protein to the domestic cat and other mammalian species [15,16,17,18]. Moreover, the obtained *CMAH* sequences were nearly identical to the domestic cat when compared to the published whole-genome sequences of these non-domestic felid species. 

Variants in the CMAH sequence can lead to alteration of protein sequence and enzyme activity. Reduction in or loss of CMAH activity disables the conversion of NeuAc to NeuGc. As a consequence, the blood type is switched from type *A* to *B* or *C*. Our recent genotyping survey for the four *CMAH* variants commonly responsible for blood type *B* (NeuAc) and *C* (both NeuGc and NeuAc) in domestic cats (namely *c.179G>T*, *c. 268T>A*, *c.364C>T*, and *c.1322delT*) did not detect associated genotypes for types *B* and *C* in any non-domestic felids [10]. In the current study, the exonic and adjacent intronic *CMAH* sequencing of 15 non-domestic felid species confirmed the lack of these four as well as any other *CMAH* variants previously implicated in blood type *B* or *C* in domestic cats [6,7]. However, other *CMAH* variants were detected in non-domestic felids; these were further examined, and one appears likely to be responsible for type *B* and/or *C* in non-domestic felids.

While 15 *CMAH* missense variants were discovered among the 15 wild felid species sequenced, only two *CMAH* missense variants were found homozygously in the cats of the two felid species with type *B* or *C.* The 635G>C variant was present in all cheetahs and cougars (all having type *B* or *C*), while the 1567A>G variant was only seen in cougars. Thus, the *CMAH* 635G>C variant, either alone or in combination with the 1567A>G variant, common to all type *B* and *C* non-domestic cats, may be responsible for type *B* or *C* in these species.

The *CMAH* 635G>C variant causes alteration of the amino acid sequence by replacing glycine with alanine (p.Gly212Ala). This exchange is also seen in all publicly available amino acid sequences of cheetahs and cougars. The identified *CMAH* variants reported here have not been reported in other species, but the 635G>C variant resides in the UlaG domain, which could affect CMAH enzyme activity. However, the protein impact programs used here did not call for any deleterious effects of the variants on the CMAH enzyme. Unfortunately, no CMAH expression or enzyme activity studies were performed here or in any other prior studies related to feline blood types. We recognize that there might be other upstream and intronic *CMAH* variants present affecting CMAH enzyme activity, and hence, the one or two promising *CMAH* variants may only be markers rather than causative variants for the non-domestic species with type *B* or *C*. 

Felid taxonomy has undergone many changes and reclassification throughout time. Although it may not be immediately obvious based upon habitat and visual appearance, it is now accepted that cougars and cheetahs are within the same genera [19,20]. Thus, the common CMAH variant may either be a marker of the genera and/or also be determining the type *B* and potentially type *C*. The limited number of animals, especially for some rare species like fishing cat, examined in this survey does not allow a definite answer. The type *C* in cheetahs as well as in one leopard and one Canada lynx may represent phenotypic errors, which are discussed in a previous publication (10). Other reasons may be found in unidentified CMAH variants or other genetic and/or environmental factors. 

## 5. Conclusions

In this study, a variant in the CMAH gene (c.635G>C) is described as the potential cause of blood type *B* in cheetahs and cougars. Due to the encountered difficulties with phenotypically typing non-domestic cats by different immunological methods and with different reagents [10], the DNA markers present in cheetahs or cougars may be diagnostically valuable to assure *A-B* matching when having to transfuse or breed a non-domestic felid and thereby avoid acute or delayed hemolytic transfusion reactions and neonatal isoerythrolysis.

## Figures and Tables

**Table 1:** **Missense variants found in *CMAH* sequence of 15 non-domestic felid species compared to sequence of *Felis catus*** (EF127684.1). Blue: found only in one species. Yellow: candidate variant for blood type *B*.

		Exon 2							Exon 4	Exon 6	Exon 9	Exon 10		Exon 11	Exon 13	Exon 14
		26C>T	50T>C	53A>G	131G>A	155A>T	160G>A	161T>C	327C>A	635G>C	1013A>G	1157A>G	1237A>G	1353G>T	1567A>G	1714T>A
Species	Blood Type	Thr9Met	Pro17Leu	Glu18Gly	Ser44Asn	Lys52Met	Val54Met	Val45Thr	Asp109Glu	Gly212Ala	Lys338Arg	Asn386Ser	Lys413Glu	Glu449Asp	Ile523Val	Ser572Thr
*Panthera tigris*	*A*	CC	CC	AA	GG	AA	AA	TT	AA	GG	AA	AA	AA	TT	AA	TT
*Panthera onca*	*A*	CC	CC	AA	GG	AA	AA	TT	AA	GG	AA	AA	AA	TT	AA	TT
*Prionailurus viverrinus*	*A*	CC	CC	AA	AA	AA	AA	TT	AA	GG	AA	AA	AA	GG	AA	TT
*Puma concolor*	*B*	CC	TT	AA	AA	AA	GG	TT	AA	CC	AA	AA	AA	GG	GG	TT
*Felis margarita*	*A*	CC	TT	AA	GG	AA	GG	TT	CC	GG	GG	AA	AA	GG	AA	TT
*Uncia uncia*	*A*	CC	CC	GG	GG	AA	AA	TT	AA	GG	AA	AA	AA	TT	AA	TT
*Felis manul*	*A*	CC	TT	AA	AA	AA	GG	TT	AA	GG	AA	AA	AA	GG	AA	TT
*Panthera leo*	*A*	CC	CC	AA	GG	AA	AA	CC	AA	GG	AA	GG	AA	TT	AA	TT
*Acinonyx jubatus*	*C*	CC	TT	AA	AA	AA	GG	TT	AA	CC	AA	AA	AA	GG	AA	TT
*Acinonyx jubatus*	*B*	CC	TT	AA	AA	AA	GG	TT	AA	CC	AA	AA	AA	GG	AA	TT
*Panthera pardus*	*A*	CC	CC	AA	GG	AA	AA	TT	AA	GG	AA	AA	AA	TT	AA	TT
*Felis nigripes*	*A*	TT	TT	AA	GG	AA	GG	TT	AA	GG	AA	AA	AA	GG	AA	TT
*Leptailurus serval*	*A*	CC	TT	AA	AA	TT	AA	TT	AA	GG	AA	AA	GG	GG	AA	AA
*Lynx rufus*	*A*	CC	TT	AA	AA	AA	GG	TT	AA	GG	AA	AA	AA	GG	AA	TT
*Neofelis nebulosa*	*A*	CC	TT	AA	AA	AA	GA	TT	AA	GG	AA	AA	AA	TT	AA	TT
*Lynx canadensis*	*A*	CC	TT	AA	AA	AA	GG	TT	AA	GG	AA	AA	AA	GG	AA	TT

**Table 2:** Distribution of variant c.635G>C in 138 samples of 15 felid species correlated to blood type *A*, *B*, or *C*.

Species	Blood Type	635GG	635CC
*Acinonyx jubatus*	*B*	0	3
*Acinonyx jubatus*	*C*	0	36
*Panthera leo*	*A*	26	0
*Panthera tigris*	*A*	19	0
*Lynx canadensis*	*A*	10	0
*Lynx canadensis*	*C*	1	0
*Uncia uncia*	*A*	10	0
*Puma concolor*	*B*	0	6
*Leptailurus serval*	*A*	5	0
*Neofelis nebulosa*	*A*	4	0
*Panthera onca*	*A*	4	0
*Prionailurus viverrinus*	*A*	4	0
*Felis manul*	*A*	3	0
*Lynx rufus*	*A*	3	0
*Panthera pardus*	*A*	1	0
*Panthera pardus*	*C*	1	0
*Felis margarita*	*A*	1	0
*Felis nigripes*	*A*	1	0

## Data Availability

Data are contained within the article. Raw data are available upon request.
